# Transport, Metabolism, and Function of Thyroid Hormones in the Developing Mammalian Brain

**DOI:** 10.3389/fendo.2019.00209

**Published:** 2019-04-03

**Authors:** Barbara K. Stepien, Wieland B. Huttner

**Affiliations:** Max Planck Institute of Molecular Cell Biology and Genetics, Dresden, Germany

**Keywords:** thyroid hormones, neocortex, prenatal development, embryonic brain development, mammalian brain development, mammalian brain evolution, hypothyroidism

## Abstract

Ever since the discovery of thyroid hormone deficiency as the primary cause of cretinism in the second half of the 19th century, the crucial role of thyroid hormone (TH) signaling in embryonic brain development has been established. However, the biological understanding of TH function in brain formation is far from complete, despite advances in treating thyroid function deficiency disorders. The pleiotropic nature of TH action makes it difficult to identify and study discrete roles of TH in various aspect of embryogenesis, including neurogenesis and brain maturation. These challenges notwithstanding, enormous progress has been achieved in understanding TH production and its regulation, their conversions and routes of entry into the developing mammalian brain. The endocrine environment has to adjust when an embryo ceases to rely solely on maternal source of hormones as its own thyroid gland develops and starts to produce endogenous TH. A number of mechanisms are in place to secure the proper delivery and action of TH with placenta, blood-brain interface, and choroid plexus as barriers of entry that need to selectively transport and modify these hormones thus controlling their active levels. Additionally, target cells also possess mechanisms to import, modify and bind TH to further fine-tune their action. A complex picture of a tightly regulated network of transport proteins, modifying enzymes, and receptors has emerged from the past studies. TH have been implicated in multiple processes related to brain formation in mammals—neuronal progenitor proliferation, neuronal migration, functional maturation, and survival—with their exact roles changing over developmental time. Given the plethora of effects thyroid hormones exert on various cell types at different developmental periods, the precise spatiotemporal regulation of their action is of crucial importance. In this review we summarize the current knowledge about TH delivery, conversions, and function in the developing mammalian brain. We also discuss their potential role in vertebrate brain evolution and offer future directions for research aimed at elucidating TH signaling in nervous system development.

## Introduction

Thyroid hormone (TH) signaling is an ancient regulatory mechanism dating back to early eukaryotes. The use of iodinated amino acids and bona fide THs to control development and trigger major life transitions precedes the ability to produce these molecules internally (1–4). Endogenous TH production within a specialized gland of animals appears in the evolution of basal chordates ~550 million years ago (1, 2, 4–6). In vertebrates THs are crucial for both development and adult life as they regulate tissue differentiation, maturation and whole body metabolic function (7). They also trigger major life transitions and metamorphosis in multiple chordate species (6, 8).

Although attempts to treat goiter with iodine-rich foods were made already in antiquity (9), the importance of thyroid gland secretions in human health was scientifically recognized only at the end of 19th century. In that time thyroid deficiency was linked to myxedematous cretinism with the first successful treatment by thyroid extract injection published by the end of the century (10, 11). THs were subsequently identified as active components, chemically characterized and synthesized in the early 20th century (12–14). Specific functions of TH signaling in brain development were also recognized with the systematic observations of the neurological cretinism prevalent in regions with iodine deficiency (15, 16). Since then our knowledge about the many roles of THs in the regulation of fetal brain development has grown exponentially. This review focuses on the functions of THs in early development of the mammalian central nervous system (CNS), with an emphasis on cerebral cortex development and evolution. Functions of THs in the postnatal development and brain function, including as regulators of adult neurogenesis, have been reviewed elsewhere (17–20).

## Production and Metabolism of THs—Maternal and Fetal Sources

Mammalian THs are produced in two forms – 3,3′,5-triiodothyronine (T3) and 3′,5′,3,5-tetraiodo-L-thyronine (T4 or thyroxine). T4, the main product of thyroid gland secretion, has a low affinity for nuclear TH receptors (TRs) and therefore is thought to act largely as a prohormone in the classical TH signaling pathway (8). In contrast, biologically active T3 has a high affinity for nuclear TRs (21, 22) and is produced by either the thyroid gland or locally from T4 by target tissues and cells (23–25). Additionally, multiple TH-derivatives arise as products of TH metabolism, some of which have biological activity while others are degradation byproducts and storage forms (26).

There are two main periods in prenatal development of placental mammals with regard to TH production and delivery into the fetal nervous system. In early development an embryo relies solely on the maternal source of THs as its thyroid gland is not yet fully functional. The thyroid gland develops early in pregnancy from an anterior region of the embryonic gut, however, in humans it does not secrete significant TH levels until mid-gestation (27). Therefore the 1st trimester of human pregnancy proceeds with a full dependence on maternal TH secretion, and afterwards fetal TH production raises gradually (28, 29). In agreement with the fetal demand for THs in pregnancy total maternal T4 and T3 levels rise through the 1st trimester and stay elevated for the remainder of pregnancy. In the same time, due to the increased binding to rising levels of maternal serum thyroxine-binding globulin (TBG), free T4 and T3 levels decrease after the initial peak at the onset of pregnancy and remain comparable with non-pregnant women (30). During pregnancy, high total TH levels are needed to meet the rising demands of the fetus as well as the mother (29, 31, 32). In cases of fetal TH production deficiencies caused by events like thyroid gland agenesis, maternal THs are largely able to substitute for fetal TH production (33, 34). Even after the onset of fetal TH production the maternal source of THs seems to be important for proper brain development, as can be deduced from the developmental deficits seen in premature infants (35). Although in the fetus total T4 and T3 concentrations are very low in early pregnancy, free T4 concentrations in the amniotic fluid and fetal serum increase to almost adult levels by mid-gestation, likely due to a low presence of TH binding carrier proteins, and could therefore exert biological function (29, 31). Free T4 is taken up by fetal tissues and gets converted to T3 locally (36).

T3, T4 and some of their metabolites are subject to the activity of three selenocysteine-containing iodothyronine deiodinases (Dio1-3) that produce both active and inactive products, thereby controlling the amount of biologically active THs and targeting their metabolites for further degradation and clearance (37). Type III iodothyronine deiodinase (thyroxine 5-deiodinase, Dio3) robustly catalyzes inner ring deiodination (IRD) of T4 and T3 to rT3 (3,3′,5′-triiodothyronine) and 3,3′-T2 (3,3′-diiodothyronine), respectively (38), resulting in inactivated forms of these hormones that have little affinity for nuclear TRs and undergo rapid removal (39). In contrast, Dio2 (type II iodothyronine deiodinase) primarily activates T4 by converting it to the active receptor-binding T3 form by outer ring deiodination (ORD) (40). Dio1 (type I iodothyronine deiodinase) can catalyze both IRD and ORD, which leads to T4 inactivation or activation, respectively, but with lower activity toward T4 than Dio2 (41). It is mainly expressed postnatally and outside of the placenta or CNS, which make it less important for fetal brain development (42, 43).

**Figure 1:**
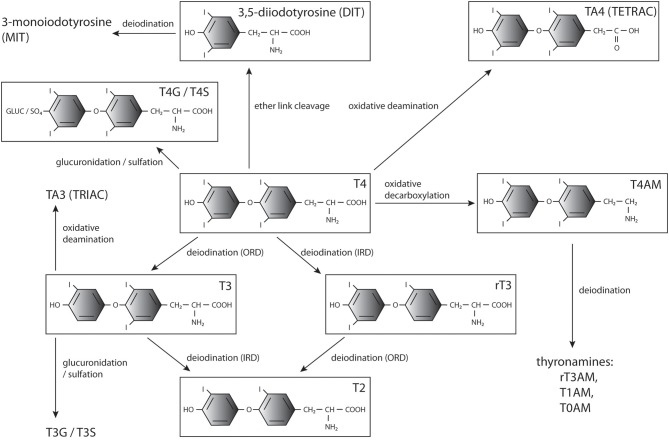
THs and major products of their metabolism. T4, 3′,5′,3,5-tetraiodo-L-thyronine (thyroxine); T3, 3,3′,5-triiodothyronine; T2, 3,3′-diiodothyronine; rT3, 3, 5′,3′,-triiodothyronine;, diiodotyrosine; MIT, monoiodotyrosine; T3G, triiodothyronine glucuronidate; T4G, thyroxine glucuronidate; T3S, triiodothyronine sulfate; T4S, thyroxine sulfate; TRIAC, triiodothyroacetic acid; TETRAC, tetraiodothyroacetic acid; T1AM, 3-iodothyronamine; T0AM, thyronamine.

In addition, TH modifications, including decarboxylation, deamination, ether-link cleavage, sulfation, and glucuronidation, affect their bioactivity and downstream metabolism (Figure 1). Most of them lead to deactivation and eventually degradation of THs (26), however some of the generated compounds, such as rT3 (44, 45), iodothyroacetic acids (tetrac and triac) and thyronamines (46–50), have been shown to convey biological effects in specific contexts. The conversions and main metabolites of THs are shown in Figure 1.

Sulfation and glucuronidation of the phenolic 4′-hydroxyl group of THs are considered phase II detoxification reactions as they increase the solubility of the products (51, 52). Sulfation is catalyzed by cytoplasmic sulfotransferases (SULTs) that transfer a sulfate group from the donor 3′-phosphoadenosine-5′-phosphosulfate (PAPS) to their substrates (53) and is utilized to inactivate THs. T3 sulfate (T3S) does not bind TRs (54) and Dio1-mediated ORD of T4 sulfate (T4S) is blocked while simultaneously IRD of both T4S and T3S is stimulated (55–58). Normally levels of sulfated THs in circulation and in excretions are low due to fast deiodination and clearance, but high levels of these metabolites are present in fetal circulation, likely due to the absence of Dio1 activity (59–62). Sulfotransferases producing T4S and T3S are present in the placenta, and sulfated TH metabolites can be transferred from the fetus into maternal circulation, potentially playing a role in regulating TH levels (52). Sulfated as well as glucuronidated THs may also serve as a pool of inactive hormones that can be mobilized by bacterial sulfatase or β-glucuronidase activity and reabsorption from the bile in the intestine (63–69) or hydrolysis by tissue sulfatases in the brain, kidneys and liver (70, 71).

## TH Delivery into the Developing Brain—Transport Across Biological Barriers

TH delivery into the fetal brain requires passage through multiple barriers at the feto-maternal interface and between fetal circulation and the CNS. THs are actively transported across tissue barriers, including placenta, and brain blood barrier (BBB), and into target cells. In circulation free THs are present only in minute amounts and mostly are bound to carrier-proteins. The main TH binding proteins in human plasma are mammalian-specific TBG, albumin and transthyretin (prealbumin, TTR) (72), the latter being also an exclusive TH carrier in the cerebrospinal fluid (CSF), where it makes up to 20% of total protein (73–75). A minor portion of THs is bound to ApoB100 and other lipoproteins (76). Carrier binding determines the amount of free *vs*. total THs in circulation, from which only the free fraction is readily available for uptake by cells, whereas protein bound THs are considered to be biologically inert (77, 78). TH entry and exit from cells are mediated by membrane transporters. A number of proteins capable of TH transport have been identified, including monocarboxylate transporters MCT 8 and 10, organic anion carrier transporter polypeptides (OATPs), Na+/taurocholate co-transporting polypeptide NTCP, and heterodimeric amino acid transporter (HAT) members/L-type aromatic and large branched-chain amino acid transporters LAT1 and 2. They differ in expression pattern and affinity for THs and their metabolites as well as ability to transport other compounds. Multiple TH transporters are expressed already during fetal nervous system development, the most important being MCT8 and OATP1C1 (79–101).

Before the onset of fetal TH production THs enter fetal tissues by passing through the placenta, which serves as an active filter allowing only limited amounts of the active hormone to enter the fetus (31, 34). The main deiodinase expressed in the placenta is Dio3 (102), the ability of which to inactivate THs is thought to protect the developing fetus from toxic levels of the maternal hormones, especially in the brain, which is uniquely vulnerable (103–105). Notably, Dio3 has a preference for T3 as substrate, which contributes to T4 being the main TH passing through the placenta (106). Dio2 is also present in the placenta, albeit at lower levels than Dio3 (107, 108), and is thought to act as a provider of bioactive T3 for local use. Total fetal T4 is kept lower than the adult level for the entire gestation in both human and rodents until birth or at 2 weeks postnatally, respectively (32, 109). An additional mechanism balancing active TH levels involving sulfation was postulated (52), although only low activity toward THs by the placental sulfotransferases was detected (110).

In the 1st trimester of pregnancy most of the THs are thought to be taken up by the fetus from the coelomic and/or amniotic fluid, while from the 2nd trimester onwards direct transfer to the fetal circulation starts to play a more important role (29). Prior to neural tube closure THs can access the developing CNS directly from the amniotic fluid. Afterwards THs get delivered into the brain either through the BBB of the developing vasculature or the choroid plexus (CP) and cerebrospinal fluid (CSF) system. Endothelial cells of both the brain capillaries and the CP express transporters and TH modifying enzymes controlling TH levels entering the brain (111).

## Cellular Signaling of THs and Its Functions in Mammalian Fetal Brain Development

### Expression and Signaling Pathways of TH Receptors in the Early Nervous System

THs versatile functions are dependent on cellular responses mediated by their interaction with various receptors expressed in cell- and tissue-specific manner. In target cells THs trigger either genomic responses mediated by DNA-binding nuclear TRs or non-genomic responses by alternative non-nuclear receptors. Genomic effects on gene transcription require members of the nuclear hormone receptor superfamily type II, in mammals encoded by two related genes arising from whole genome duplication in vertebrates: THRA/NR1A1 and THRB/NR1A2, which produce TR α and β, respectively (112–114). Each of these genes can undergo alternative splicing and harbors alternative promoters, resulting in a number of distinct isoforms differing in their ability to bind target DNA sites, ligand binding, and co-factor recruitment (114, 115). The isoforms that possess both DNA and ligand binding capacity and localize to the nucleus are TRα1 and β1-3 (with TRβ3 being rat-specific), and these are the ones that mediate the genomic effects of THs (116, 117). Other isoforms act as dominant-negative regulators or have non-genomic functions (118–120). TRβ1 and 2 possess the same DNA binding domain, but their N-termini differ in the activation domain, which in β2 favors coactivator recruitment (121, 122). TRα1 and TRβ1 differ in DNA-binding affinity and selectivity (123), T3 affinity (124), and the ability to form dimers (125). T3 is the active form of the hormone capable of binding to these receptors as T4 has about 10 times lower affinity for TRs (21, 22). However, direct T4 binding with biologically significant effects has also been shown recently (126, 127).

To affect transcription of target genes TRs bind DNA as either homodimers or heterodimers with retinoid-X-receptors and recognize TH response elements (TREs) in promoter regions of regulated genes (114). TRs lacking bound THs can bind DNA as aporeceptors, which represses target gene transcription by recruiting corepressor complexes with histone deacetylase activity (128, 129). T3 binding lifts this repression and leads to target gene transcription, which is necessary for normal nervous system development (130–132). While T3/TR interaction results in coactivator recruitment, chromatin restructuring, and transcriptional activation for most targets, some genes can also be repressed by TRs with bound THs (133, 134). Accordingly, a meta-analysis study of genes transcriptionally regulated by THs in the nervous system identified over 700 curated targets, however the extent and mode of their regulation is likely to differ during development and in specific cell types (135). More targeted studies are needed to explain the differential cellular responses to THs in various contexts. The interplay between various TR isoforms, chromatin re-modeling and transcriptional machinery leads to complex tissue and cell-specific responses in various contexts and comprehensive reviews on the mechanistic aspects of the genomic pathway are available (136–138).

Tissues differ in TR isoform expression patterns and cell-specific functions. TR isoforms share many common targets, however, there is marked spatiotemporal variation in the degree and mode of regulation and target overlap. Frequently cells express multiple isoforms with distinct roles arising due to differences in the respective protein levels or intrinsic activity (117). Nuclear TRs are expressed in the developing brain of humans and rodents (22, 139, 140), and T3 binding in the human brain occurs even before fetal thyroid gland maturation (22, 141, 142). TRα1 is the major isoform expressed in neurons from early fetal development in humans and rodents onwards, while TRβ increases perinatally and is more abundant in specific neuronal types such as hippocampal pyramidal and granule cells, paraventricular hypothalamic neurons and cerebellar Purkinje cells (143–145). Interestingly, TRβ1 is also expressed in the germinal zones of cerebral cortex (145). During early postnatal development in rodents TRβ is specifically required for enhancing the expression of the striatum-enriched gene *Rhes* (146). *Rhes* functions in multiple signaling pathways and has been implicated in the regulation of dopamine-mediated synaptic plasticity of striatal neurons, in striatum-related behaviors, and in neurodegeneration in the course of Huntington disease (147). Moreover, TRβ1 and 2 are required for the cochlear and retina development, and TRβ null mice have defects in auditory and visual development (148). TRβ2 also plays a role in establishment and maintenance of the hypothalamus-pituitary-thyroid gland axis (114). Most neurons express both TRα and TRβ receptors, however, the relative expression levels differ, which can have important functional consequences such as in the hippocampus, where TRα but not β is necessary for proper GABAergic interneuron innervation and behavior (145, 149). The relative abundance of both receptors was also proposed to control proliferation/differentiation balance in the developing brain (145). In addition, certain specific cell types express exclusively either TRα or TRβ form. For instance, parvalbumin (PV) positive cells in the CA1 of the hippocampus express preferentially TRα while the PV^+^ interneurons in the somatosensory cortex produce mostly TRβ (149). Also developing cerebellar granule cells express TRα1 but not TRβ while Purkinje cells produce mostly TRβ (144, 145, 150).

TR mutations in both rodents and humans have been linked to a range of behavioral and cognitive phenotypes, including changes in sensory, attention, emotion and memory functions, but their effects are complex and usually more benign than those of hypothyroidism (149, 151–155). Detrimental effects of hypothyroidism are thought to occur largely due to the repressive activity of TRs lacking bound THs, as mice completely lacking both TR receptor types are viable and without major defects (153). Moreover, TRα1 KO rescues the viability of Pax8 KO mice, which present with thyroid agenesis and lethal congenital hypothyroidism during the early postnatal period (156), and partly rescues the Dio3 KO phenotype (157). TRs lacking bound THs are generally implicated in maintaining the proliferative, undifferentiated state of neural progenitors, while T3-bound receptors promote transcription of genes triggering cell differentiation and maturation (129, 158–160).

In addition to the classical pathway mediated by nuclear TRs, a growing list of TH effects have been linked to their non-genomic actions, including regulation of actin polymerization (161), Dio2 activity (162), ion transport (163), Akt/PKB and mTOR pathway activation (164), and fatty acid metabolism (165). Non-genomic effects of THs can also influence cell proliferation and survival (166). Among receptors mediating the non-genomic functions of THs is a cell surface TH receptor, integrin αvβ3 (167, 168), which preferentially binds the T4 pro-hormone to activate the MAPK signaling cascade. This interaction promotes angiogenesis (167) and proliferation in osteoblasts and various cancer cell types (169–171). Signaling through this receptor has also been implicated in neocortical development as T4 binding to integrin αvβ3 upregulates progenitor proliferation in this structure (172). A detailed review of the non-genomic effects of THs in various cell types can be found elsewhere (120).

THs also interact with other signaling pathways during cortical development. In neural development sonic hedgehog (Shh) signaling leads to an increase in Dio3 expression while decreasing Dio2 by ubiquitination (108). In turn both fetal and adult brain T3 upregulated Shh production (134, 173), thus providing a negative feedback loop. TH and Shh pathways interact also in cerebellar development to control granule cell precursor proliferation (174). Brain morphogen retinoic acid (RA) shares common carrier proteins with THs, and their nuclear receptors dimerize. RA can also increase MCT8 expression to increase TH import (175). Another transcription factor, COUP-TF1 (Chicken Ovalbumin Upstream Transcription Factor 1), has been shown to bind to DNA sites overlapping with TREs and to block TR access and activation (176, 177) thereby modulating TH signaling. Genes that show the presence of both TR and COUP-TF1 binding elements include calcium calmodulin-dependent kinase IV (CamKIV) (177, 178), which is important for both GABAergic and glutamatergic neuron production (179, 180). Emx1 and Tbr1 genes are also controlled by both THs and COUP-TF1, with the latter factor modulating the timing and magnitude of the T3 response (180). Similarly, nuclear liver X receptor β interacts with TH signaling in regulating cortical layering, likely by influencing the expression of their common target, the reelin receptor ApoER2 (181).

### Developmental Hypothyroidism and Its Impact on Brain Development

The complexity of TH production, delivery, and metabolism contributes to varying clinical presentations of different TH signaling deficiencies during gestation, with the most severe being iodine deficiency which impairs both maternal and fetal TH supply (15, 182). Maternal iodine deficiency or severe hypothyroxinemia alters embryonic brain development even before the fetal thyroid gland becomes functional (183, 184), and leads to profound neurological cretinism with defects in sensory, motor and cognitive functions (15, 28, 185, 186). TH deficiencies, even when limited to the 1st trimester of gestation, are linked to cognitive deficits and neurodevelopmental delay (183, 187, 188). In contrast, fetal TH production defects, such as congenital hypothyroidism caused by thyroid agenesis, can largely be compensated by maternal THs (33, 34, 189), with most deficiencies in development arising postnatally if these defects are not treated (109, 190).

Given the selective placental permeability for T4, even mildly hypothyroid or asymptomatic cases of maternal iodine deficiency, lowering T4 but not T3 levels, can reduce fetal THs enough to cause developmental defects (182, 186, 189). Moreover, maternal T4 but not T3 supplementation protects the brain from hypothyroid injury until birth (34, 189, 191). As in the placenta, the main TH form transported into the CNS is T4, and the majority of the cerebral cortex T3 comes from local tissue production by Dio2 (192, 193), rendering the brain dependent mostly on circulating fetal T4 levels (28). The brain seems to be privileged in taking up T4 from the fetal circulation compared to other tissues, while the opposite is true for T3 (34). TH transporters facilitate entry from the circulation into the developing brain. Postnatally T4 is mainly taken up by astrocytic OATP1C1 and converted to bioactive T3 by the action of Dio2 (94, 194), which is expressed almost exclusively in glial cells (195, 196). Generated bioactive T3 is then provided to neurons, which lack Dio2 activity but express high levels of Dio3, allowing them to deactivate glia-derived excess THs (43, 196, 197). Neurons take up T3 preferentially over T4 via the MCT8 transporter either from astrocytes or directly from the interstitial fluid (198–200).

The Dio2/Dio3 activity balance provides an important mechanism for regulating active T3 levels in the brain to protect against excess THs (201). Both Dio2 and Dio3 activities are present in the fetal brain already from the 1st trimester onwards but show opposing trends with Dio3 being more active early and Dio2 toward the end of gestation (202–204). Dio3 KO in mouse, in contrast to other deiodinases, causes widespread abnormalities in brain and sensory organs, but it is unclear to which degree this phenotype is generated prenatally and arises due to placental or CNS deficiency (104, 105, 205). Similarly, human mutations affecting Dio3 imprinting result in Temple or Kagami-Ogata syndromes that impair brain function; however, whether this phenotype can be fully attributed to the altered dosage from the Dio3 locus is unclear (206). Additional mechanisms controlling active TH levels may also be present as TH sulfotransferases were shown to be expressed and active in the developing human brain (207, 208).

**Figure 2:**
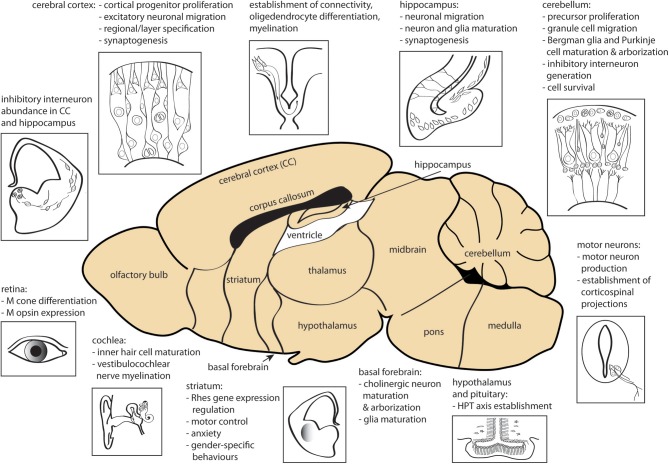
Sites of action of THs during CNS development. Processes affected by TH signaling prenatally and in early postnatal development are shown. CC, cerebral cortex; HPT, hypothalamus-pituitary-thyroid gland; M, middle-wavelength sensitive. The figure was created using the mouse brain schematic available under Creative Commons CC0 1.0 Universal (CC0 1.0) Public Domain Dedication license.

Fetal and perinatal TH deficiency, due to congenital hypothyroidism or iodine deficiency, has a dramatic negative impact on cerebral development, affecting multiple regions including cerebral cortex, hippocampus, amygdala, and basal ganglia as well as motor neurons, cochlea, retina and interregional connectivity (15, 183, 184). Most of the early brain developmental events (proliferation of neural progenitors and neuronal migration in the neocortex, hippocampus, and medial ganglionic eminence) occur before fetal TH production, and thus are predominantly under the control of maternally-derived TH signaling. However, later stage processes (ongoing neurogenesis and migration, axon growth, dendritic arborization, synaptogenesis, and early myelination) occur after the onset of fetal TH production and proceed under the control of both fetal and maternal THs. Further brain developmental events (cortex pyramidal cell, hippocampal granule cell and cerebellar granule and Purkinje cell migration, gliogenesis, and myelination) occur postnatally and are therefore controlled entirely by neonatal THs. TH signaling has an effect on all of these processes (158, 209). The diverse actions of TH in early brain are summarized in Figure 2.

### Functions of TH Signaling During Development of Mammalian CNS

Human cretinism has been extensively modeled in rodents. In human, cortical neurogenesis occurs between week 5 and 20 of gestation, which is the period when the fetus depends primarily on the maternal source of THs, corresponding roughly to rat E12-18 (27, 209). In cortical development neurons are generated from progenitor cells residing in the subventricular zone and migrate basally along radial glia fibers to form an ordered 6-layered cortical plate, a process controlled largely by pioneer Cajal-Retzius and subplate neurons (210, 211). Perturbations of this migratory process lead to defects in cortical morphology and function (212). Even mild or transient maternal hypothyroxinemia during neurogenesis retards fetal glutamatergic neuron migration along the radial glia scaffold in the rat sensory cortex and hippocampus, without affecting tangentially migrating GABAergic neurons. This deficiency results in reduced neocortical thickness, blurred cortical layering and subcortical band heterotopia, likely responsible for increased seizure susceptibility and altered behavior (184, 190, 213–216). Improper neuronal migration also leads to alterations in callosal connectivity (213, 217). These migration defects can be at least partly attributed to a direct effect of the lack of THs on guiding cues as THs regulate Reelin, Dab1, and Vldlr expression in rat neocortex and cerebellum (218–220). T3 signaling also controls the expression of lipocalin-type prostaglandin D2 in Cajal-Retzius cells and hippocampal neurons during development (221), a protein known to affect glial cell migration (222). Moreover, a large subset of subplate neuron-enriched genes were shown to be under TH regulation (160). Maternal hypothyroidism alters gene expression in the brain by midgestation, and while it can be corrected by T4 application (223), the morphological changes persist if hormones are replaced after the critical window has closed (36).

TH signaling affects not only migration but also enhances progenitor proliferation and cortical neurogenesis, which is regulated by both genomic and non-genomic TH action (172, 180, 224). Hypothyroidism causes cell cycle disruption, increased apoptosis and reduction in both apical and basal progenitor pools and defects in neuronal differentiation, leading to cortical thickness reduction and decreased neuron number, especially in upper cortical layers (224). THs were shown to upregulate genes involved in cell cycle regulation and sustained proliferation in the developing cortex, such as POU2F1/Oct-1 or Nov (178, 223). Signaling through various pathways could have opposing roles in regulating proliferation/differentiation balance as T4 binding to integrin αvβ3 upregulates progenitor proliferation in the developing cortex (172), while T3 regulates gene expression in primary cerebrocortical cells via a nuclear TR-dependent pathway consistent with a role in promoting neuronal differentiation (160). Even mild hypothyroxinemia induces shifts in gene expression in developing hippocampus and neocortex (225). Among TH-regulated targets are genes involved in neuronal specification and function, such as Emx1 (Empty spiracles homolog 1), Tbr1 and neurogranin (180, 226–228), as well as cytoskeleton components and ECM molecules, which impact on both proliferation and neuronal migration (134, 229). T3 also regulates the expression of DNA methyltransferase Dnmt3a in mouse brain, potentially extending the genomic effects of TH action beyond directly regulated genes by affecting global DNA methylation states (230). Seemingly contradictory functions of THs in promoting progenitor proliferation and neuronal differentiation may stem from specific spatiotemporal expression of their transporters, metabolizing enzymes, and effectors that mediate different actions in various cell types in the course of development.

While progenitor proliferation, cortical neurogenesis and early neuronal migration occur largely prenatally, THs have a profound effect also on perinatal CNS developmental events. During that period, the TH deficiency associated with congenital hypothyroidism leads, in both rodent and humans, to defects in late neuron migration, cerebellar neuron and glia arborization and maturation (231–233), astrocyte and neuron differentiation in hippocampus (234–236), inhibitory neuron development and function (237, 238), oligodendrocyte differentiation and myelination (129, 239, 240), and synaptogenesis (241, 242). TH signaling also controls spinal motor neuron generation in vertebrates (243) and establishment of corticospinal projections (244).

The impact of perinatal TH deficiency on brain development has been intensely studied in two vital regions associated with hypothyroid injury, especially related to motor function impairment—the striatum and the cerebellum (245). In mammalian cerebellum the final TH-dependent stages of development occur perinatally, when cells from the external germinal layer proliferate and migrate to the inner granular layer forming connections with maturing Purkinje cells (246). TH signaling affects all of these processes. In cerebellum migration of granular cells requires ligand bound TRα, while maturation of Purkinje cells depends on the functions of both TRα and β isoforms. Additionally, TRβ is required for adequate granule cell proliferation (247). Interestingly, the hypothyroid injury on the developing cerebellum can be largely rescued by TRα1 deletion, in agreement with the function of TH in relieving the receptor-mediated transcriptional repression (248).

In the striatum a connection between TH-regulated gene expression and brain-region specific function involves the Ras-like GTP-binding protein Rhes/Rasd1. Despite being expressed in multiple brain regions from midgestation this gene shows a specific striatal upregulation in early postnatal rodent development that is critically dependent on THs (249–251). Developmental Rhes enrichment in this structure is dependent on T3 binding to TRβ isoform (146), however adult expression seems to rely primarily on TRα (252). Interestingly, Rhes functions in G-protein coupled receptor signaling as well as in PI3K/Akt/mTOR pathways (253, 254) to modulate synaptic transmission (255), and Rhes KO animals have deficits in striatum-controlled behaviors (256), providing a potential functional link between hypothyroidism and resulting motor and affect dysfunctions.

## THs in Mammalian Brain Evolution

In addition to their relevance regarding neurodevelopmental disorders, THs may have played a crucial role in human brain evolution. Although mostly limited to comparison between human and rodents, a number of important differences in TH signaling have been characterized. Spatiotemporal expression patterns of TH transporters are species-specific and can lead to drastic differences in TH metabolism, evident especially in disease states. Strikingly, the effects of MCT8/SLC16A2 mutations, which in human cause severe brain hypothyroidism with concomitant hyperthyroidism in circulation and peripheral organs, known as Allan-Herndon-Dudley syndrome (AHDS), characterized by severe intellectual and motor disability (257–259), are not fully recapitulated by mice, especially with regard to the neurological phenotype (91, 260–262). In rodents, only MCT8 and OATP1C1 double-inactivation causes cerebral hypothyroidism and associated defects (263). Various explanations, including the presence of compensatory alternative transport or T3 production pathways in rodents (264, 265) or the differential expression of the LAT2 transporter in neurons (91), have been suggested.

A potential evolutionary difference in TH delivery between rodents and human may exist, pertaining to the carrier protein TTR. In human TTR is present in the CSF as early as from the 8th fetal week (75), and in contrast to TBG and albumin there are no known individuals with TTR null mutations, suggesting its vital role in development (72). However, TTR null mice are viable and do not have overt symptoms of hypothyroidism in the CNS (266). Interestingly, TTR evolution in vertebrates, leading to its synthesis in the CP and a shift in specificity from T3 to T4 in the mammalian protein, coincides with the emergence of the cerebral cortex as a novel structure (72). It is tempting to speculate that the evolutionary expansion of the neocortex in the primate lineage may be linked to increased dependence on the function of TTR during development. Subtle differences in serum TTR abundance and posttranslational modifications were detected between human and several other species of great apes, but their functional and evolutionary importance remains to be elucidated (267).

In rodent neocortex development increasing TH-mediated integrin αvβ3 activation promotes basal progenitor proliferation (172). In contrast, blocking integrin αvβ3 has the opposite effect on ferret basal progenitors (268). Increased pool size and proliferative capacity of basal progenitors are thought to have contributed to the evolutionary expansion of the neocortex, especially in the primate lineage (229). Interestingly, a number of human genes implicated in TH metabolism are altered in human basal progenitors compared to mouse (208), which may affect the magnitude and timing of TH action during cortical neurogenesis.

One of the major concepts in human evolution is neoteny, especially in relation to brain development and function (269). Alterations is TH signaling are known to underlie evolutionary heterochrony in various animal species (6), including our closest living relatives, the chimpanzees and bonobos (270). The global TH status in rodents is connected to either accelerated or delayed development in hyperthyroid and hypothyroid pups, respectively (271). Given that in the CNS THs tend to accelerate cell type maturation (272, 273), one could speculate that prolonged or enhanced brain protection from THs and spatiotemporal alterations in metabolic enzyme and effector expression in the primate lineage could have delayed differentiation, contributing to human neoteny. Further studies investigating species-specific differences in TH pathways in brain development, especially including other model species, beyond human and rodent, could help to test this hypothesis.

## Conclusions

TH action with regard to mammalian brain development is highly pleiotropic, and despite many advances the complexity of their delivery, metabolism, and cell-specific responses make it difficult to dissect specific functions in brain regions and cell subtypes in the course of development. With the advent of single-cell transcriptomics and the CRISPR/Cas9 technology, the spatiotemporal dissection of TH signaling in various cell types across the nervous system should become faster and more precise. This is of crucial importance, as in addition to the long-recognized role of TH deficiency in neurodevelopmental defects, undiagnosed developmental hypothyroxinemia may be linked to common neurological disorders such as ataxias and epilepsy (274, 275). Elucidation of the mechanisms underlying these pathologies down to the cellular and subcellular level could aid better diagnostic and therapeutic interventions. Understanding and expanding the existing catalog of the evolutionary differences in TH signaling, which momentarily includes mostly genes linked to human genetic diseases such as AHDS or Kagami-Ogata syndrome, could also contribute to the generation of better disease models. Of note, when reaching conclusions about the role of THs in the human brain from rodent studies, it is important to keep in mind the at times profound phenotypic variation across species and its impact on disease presentation and potential treatments.

## Author Contributions

BS and WH made substantial contributions to the conception and drafting of the work and revising it critically. WH approved the final version of this manuscript.

### Conflict of Interest Statement

The authors declare that the research was conducted in the absence of any commercial or financial relationships that could be construed as a potential conflict of interest.
